# Strengthening antimicrobial resistance diagnostics in National Reference Laboratories across One Health in South and Southeast Asia: impact of external quality assessment and targeted follow-up

**DOI:** 10.1093/jac/dkaf266

**Published:** 2025-07-29

**Authors:** Freshwork Ayalew Abegaz, Hiba Al-mir, Tomislav Kostyanev, Kristi Prifti, Soo-Young Kwon, Tobin Guarnacci, Lone Brink Rasmussen, Mohammad Julhas Sujan, Patrícia Teixeira Dos Santos, Rangsiya Prathan, Taradon Luangtongkum, Pattrarat Chanchaitong, Pitak Santanirand, Watcharaporn Kamjumpho, Ondari D Mogeni, Marianne Holm, Florian Marks, Rungtip Chuanchuen, Rene S Hendriksen, Nimesh Poudyal

**Affiliations:** EPIC, International Vaccine Institute, 1 SNU Research Park, 1 Gwanak-ro, Gwanak-gu, Seoul 08826, South Korea; National Food Institute, Technical University of Denmark, Kgs. Lyngby, Denmark; Department of Clinical Microbiology, University Hospital of Southern Denmark—South-West Jutland, Esbjerg, Denmark; EPIC, International Vaccine Institute, 1 SNU Research Park, 1 Gwanak-ro, Gwanak-gu, Seoul 08826, South Korea; EPIC, International Vaccine Institute, 1 SNU Research Park, 1 Gwanak-ro, Gwanak-gu, Seoul 08826, South Korea; EPIC, International Vaccine Institute, 1 SNU Research Park, 1 Gwanak-ro, Gwanak-gu, Seoul 08826, South Korea; National Food Institute, Technical University of Denmark, Kgs. Lyngby, Denmark; EPIC, International Vaccine Institute, 1 SNU Research Park, 1 Gwanak-ro, Gwanak-gu, Seoul 08826, South Korea; National Food Institute, Technical University of Denmark, Kgs. Lyngby, Denmark; Research Unit for Microbial Food Safety and Antimicrobial Resistance, Faculty of Veterinary Science, Chulalongkorn University, Bangkok, Thailand; Research Unit for Microbial Food Safety and Antimicrobial Resistance, Faculty of Veterinary Science, Chulalongkorn University, Bangkok, Thailand; Research Unit for Microbial Food Safety and Antimicrobial Resistance, Faculty of Veterinary Science, Chulalongkorn University, Bangkok, Thailand; Department of Medical Sciences, National Institute of Health, Bangkok, Thailand; Department of Medical Sciences, National Institute of Health, Bangkok, Thailand; EPIC, International Vaccine Institute, 1 SNU Research Park, 1 Gwanak-ro, Gwanak-gu, Seoul 08826, South Korea; EPIC, International Vaccine Institute, 1 SNU Research Park, 1 Gwanak-ro, Gwanak-gu, Seoul 08826, South Korea; EPIC, International Vaccine Institute, 1 SNU Research Park, 1 Gwanak-ro, Gwanak-gu, Seoul 08826, South Korea; Cambridge Institute of Therapeutic Immunology and Infectious Disease, University of Cambridge School of Clinical Medicine, Cambridge, UK; Heidelberg Institute of Global Health, University of Heidelberg, Heidelberg, Germany; Madagascar Institute for Vaccine Research, University of Antananarivo, Antananarivo, Madagascar; Research Unit for Microbial Food Safety and Antimicrobial Resistance, Faculty of Veterinary Science, Chulalongkorn University, Bangkok, Thailand; National Food Institute, Technical University of Denmark, Kgs. Lyngby, Denmark; EPIC, International Vaccine Institute, 1 SNU Research Park, 1 Gwanak-ro, Gwanak-gu, Seoul 08826, South Korea

## Abstract

**Objectives:**

The intent of this study was to evaluate and improve microbiology laboratory diagnostic capacity in selected National AMR Reference laboratories from South and Southeast Asia for AMR testing utilizing EQA and targeted follow-up support.

**Methods:**

A baseline assessment was conducted to evaluate quality management system (QMS) practices for laboratories participating in the Strengthening External Quality Assessment in Asia (EQASIA-EQA) programme for the first time. Following each EQA iteration, laboratory assessments were conducted, and underperforming laboratories received online consultations to identify root causes of deviations and implement corrective and preventive actions (CAPA). Laboratories with persistent underperformance, onsite visits and targeted training sessions were provided to support quality improvement.

**Results:**

Significant gaps were identified in the QMS of the 24 laboratories surveyed, with 38% lacking a QMS and 42% not conducting internal audits. Many laboratories lacked standard operating procedures (SOPs) for key processes, CAPA and equipment maintenance. Training and competency assessments were insufficient, with 50% of laboratories not conducting competency assessments. Additionally, 46% did not perform root cause analysis for EQA deviations, and 42% lacked a structured quality control programme. Water purity testing and equipment maintenance were also inadequate. Of the four baseline-assessed laboratories, three underperformed twice, and one underperformed three times across EQA iterations. Key deficiencies included the absence of a QMS and SOPs, as well as inadequate staff training and internal quality control. After targeted follow-up support, improvements were observed in QMS implementation, SOP development, staff training and quality control.

**Conclusions:**

Participation in EQA, combined with targeted follow-up support, enhances laboratory quality and performance. These improvements ultimately contribute to the provision of reliable data that can guide policy actions to combat AMR.

## Background

The emerging challenge of antimicrobial resistance (AMR) poses a significant threat to global healthcare. Diagnostic laboratories are at the forefront of combating this issue by accurately identifying pathogens and detecting antimicrobial resistance through antimicrobial susceptibility testing (AST).^1,2^ In low- and middle-income countries (LMICs), laboratories, however, often struggle with resource limitations, inadequate infrastructure and restricted access to laboratory strengthening efforts and technical assistance leading to challenges in implementing effective quality assurance programmes and achieving consistently high-quality test results.^3,4^ One key strategy for enhancing diagnostic performance is through laboratory participation in external quality assessment (EQA) schemes. EQA is an essential component of a laboratory quality management system (QMS), enabling laboratories to regularly check the performance of routine tests and assess the competency of technical personnel. EQA within a One Health framework allows for the assessment of human, veterinary and food safety laboratories using harmonized standardized schemes. This enhances the readiness of laboratory networks to respond to infectious diseases and AMR affecting both human and animal populations.^5,6^ Participation alone does, however, not ensure analytical quality. Laboratory management and personnel must consistently strive to enhance quality by addressing gaps identified through the application of EQA feedback and follow-up support.^7^

Strengthening laboratory services and systems is essential to address the increasing burden of diseases that are significant to global public health.^8^ The UK Aid Fleming Fund Regional Grant Strengthening External Quality Assessment in Asia (EQASIA) project, initiated in 2020 and scheduled to terminate at the end of 2025, aims to deliver EQA services for enhancing AMR diagnostic within the One Health sector across South and Southeast Asia serving National Reference Laboratories (NRLs) and Centres of Excellence (CoE) striving to improve the quality of laboratory-based surveillance for WHO Global Antimicrobial Resistance Surveillance System (GLASS) pathogens and Food and Agriculture Organization (FAO) priority pathogens.^9^

The intent of this study was to evaluate and improve microbiology laboratory diagnostic capacity in selected National AMR Reference laboratories from South and Southeast Asia for AMR testing utilizing EQA and targeted follow-up support.

## Methods

### Selection of the study sites/centres

This study focused on national AMR reference laboratories from South and Southeast Asia, selected through a structured process during the inception phase of the EQAsia project in 2020.^10^ Laboratories were primarily selected based on their designation as AMR National Reference Laboratories by national authorities or AMR coordinating committees under their National Action Plans (NAPs). Additional laboratories were identified through consultations with the Fleming Fund Country and Regional Grants, WHO GLASS, FAO networks and scoping visits conducted by Mott MacDonald.

Ultimately, laboratories from six of the seven South Asian countries (Bangladesh, Bhutan, Maldives, Nepal, Pakistan, Sri Lanka) and 8 of the 11 Southeast Asian countries (Brunei Darussalam, Indonesia, Lao PDR, Papua New Guinea, Philippines, Thailand, Timor-Leste, Vietnam) were included covering approximately 78% of countries in these two regions. This included 10 Fleming Fund priority countries and three additional countries (Brunei Darussalam, Maldives, Philippines) that were joined upon request.

Prior to each EQA cycle, coordinators circulated a pre-notification email containing key information and a sign-up link. This was distributed to a master list of laboratories and stakeholders, including representatives from Country Grants (CG), the Fleming Fund (FF) and Mott MacDonald (MM). In the first EQA iteration, 37 laboratories from 12 countries were invited, and 23 laboratories from 11 countries (Bangladesh, Bhutan, Brunei Darussalam, Indonesia, Lao PDR, Maldives, Nepal, Pakistan, Philippines, Sri Lanka and Timor-Leste) participated. Participation increased to 24 laboratories in the second iteration and continued to expand in subsequent cycles.

### EQA scheme

As part of the EQASIA project, a total of seven EQA cycles were conducted between February 2021 and November 2023; three cycles in 2021, followed by two cycles per year in both 2022 and 2023. Each iteration included WHO GLASS and FAO-priority pathogens and was designed to assess the performance of laboratories in both human and animal health sectors. Detailed results from EQA cycles 1–7, including participation, performance outcomes, panel composition, validation and evaluation methods, have been published elsewhere.^9,11^

### Selection criteria for underperforming laboratories

Underperforming laboratories were identified based on two criteria: (i) pathogen identification and (ii) AST. EQA trials 1–2 were designed purely as laboratory quality assessments, AST results were strictly scored as ‘1’ for correct or ‘0’ for incorrect. From EQA 3–7, the trials were redesigned as more clinical quality assessments complying to CLSI and International Organization for Standardization (ISO) 20776-2:2007 guidelines, categorizing results as ‘resistance’, ‘intermediate’ and ‘susceptible’ resulting in deviation scores as ‘4’ (correct), ‘3’ (minor error), ‘1’ (major error), or ‘0’ (very major error).^9^ Among those scoring below 95%, 2–6 laboratories from each EQA iteration were designated as underperforming. Due to EQASIA resource constraints, the laboratories were followed up through survey-based questionnaires and online consultation meetings.

### Procedure for the follow-up assessment

After each EQA iteration, laboratory assessments were conducted, followed by online consultations to support underperforming laboratories. Onsite visits and training were arranged for consistently underperforming labs, focusing on AST based on European Committee on Antimicrobial Susceptibility Testing (EUCAST) and CLSI guidelines, including disk diffusion, broth micro-dilution, quality control and the detection of multidrug-resistant pathogens. These efforts aimed to identify the root causes of deviations observed during the EQA trials, provide guidance on implementing corrective and preventive actions (CAPA) and foster continuous improvement in laboratory practices for pathogen identification and AST.

The first remote follow-up assessment was conducted after EQA 1 trial distribution between May and August 2021 with two animal health laboratories. It included online laboratory walkthroughs, expert interviews and remote document reviews.^12^ From the second to the seventh round of follow-up assessments, an online survey questionnaire was distributed. The elements of the survey were based on ISO 15189:2012, the CLSI^13^ and ISO 17025:2017. The questionnaire included 122 questions covering facility and safety, management, quality assurance, document control, personnel training, internal quality control, equipment maintenance, specimen handling, reagent management, water quality and IT systems (Supplementary data, available as Supplementary data at JAC Online).

To establish baseline data on laboratory quality management practices and assess compliance with ISO standards, this online assessment questionnaire was sent to 24 laboratories that participated in the second iteration of EQA and seven additional laboratories that joined the EQASIA programme in subsequent EQA iterations. A total of 24 laboratories (17 from EQA 2 and all seven new participants) completed the survey, including one laboratory that also participated in a virtual follow-up visit following the first EQA iteration. From EQA3 to EQA7, the online survey was administered to laboratories identified as underperforming in each cycle. A total of 23 laboratories completed these follow-up surveys. Among them, 18 laboratories had previously responded to the baseline survey, while five laboratories had not completed the baseline survey despite multiple reminders. Of the 23 underperforming laboratories, two underperformed in three EQA iterations (completing the follow-up survey three times), and four underperformed in two iterations (completing the survey twice).

**Figure 1.**
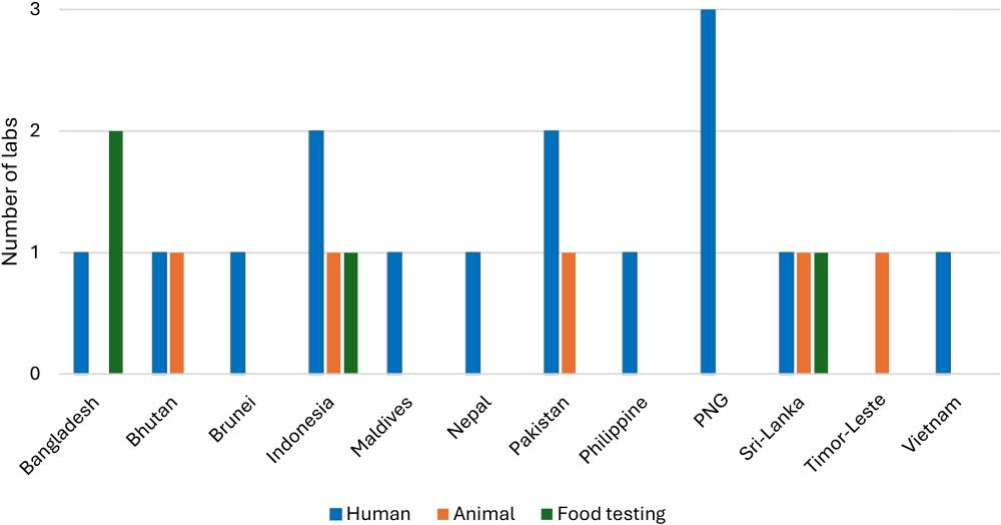
Distribution of laboratories per country and health facility from 2021 to 2023, PNG, Papua New Guinea.

Among the 24 laboratories surveyed during the baseline assessment (Figure 1), four laboratories were identified as underperforming in two to three different EQA iterations. To assess their progress in addressing the findings from the baseline assessment, identical survey questionnaires were sent to these laboratories for continued evaluation, with additional support provided as needed. Supporting documents, including quality manuals, standard operating procedures (SOPs), audit reports, quality control records, CAPA reports, equipment maintenance logs and temperature records, were requested and reviewed to verify survey responses and confirm the implementation of CAPA.

Based on the survey responses, relevant observations were summarized, and tailored recommendations were generated and shared with the impacted laboratories. Supporting documentations were also provided to help identify root causes and implement CAPA, as applicable.

Online consultation meetings were also conducted with each underperforming laboratory to discuss deviations observed in the EQA for pathogen identification, AST and quality management systems observations based upon respective survey responses. The meetings included discussions on potential root causes and providing guidance in support of the implementation of appropriate CAPA. Laboratories were contacted in advance to schedule a time for a 1 h online session. To aid in preparation, supporting documents, including a list of observations, were shared with the laboratories beforehand. During the meetings, the EQASIA team presented their findings, addressed discrepancies in the EQA trials and discussed issues related to consumables and techniques.^12^

### Data collection

An online survey was developed and administered using Survey Method (https://surveymethods.com/), a secure web-based platform for professional survey design and data collection. A single survey link was distributed via email to all participating laboratories.

## Results

Among the 24 surveyed laboratories, 16 were from the public health sector, and eight were from the animal sector including food safety laboratories.

### Gaps identified from survey responses in baseline assessment

#### Facility/safety

Of the 24 laboratories surveyed, 88% (21/24) had adequate space for performing bacterial identification and AST. A total of 63% (15/24) laboratories did not, however, control, monitor, or record environmental conditions such as room temperature and humidity, which can affect the integrity of the samples, equipment, as well as the health of the staff.

#### Organization and quality assurance/quality management

A QMS was not implemented in 38% (9/24) laboratories. Additionally, 25% (6/24) laboratories did not have a designated quality manager overseeing the laboratory operations and QMS activities. Annual reviews of the QMS and internal audits were not conducted in 42% (10/24) laboratories. Furthermore, 29% (7/24) of laboratories did not review records of errors and incident reports at defined intervals to identify trends and initiate CAPA, as applicable.

#### Document control/SOPs

Document control and the availability of up-to-date SOPs are essential components of quality management in NRLs. However, 29% (7/24) of laboratories lacked SOPs for media preparation, pathogen identification and AST. Additionally, SOPs for identifying and managing nonconformities were absent in 42% (10/24) of laboratories, and 58% (14/24) did not have SOPs for conducting internal audits.

Procedures for conducting root cause analysis and implementing CAPA were absent in 54% (13/24) of laboratories. Additionally, in 38% (9/24) of laboratories, quality management procedures, forms and records were not up to date, controlled, or approved by authorized personnel.

#### Personnel training and assessment

Personnel training and competency assessments were significant gaps. Training in assigned tasks was not provided in 33% (8/24) of laboratories. Competency assessments were not demonstrated in 50% (12/24) of laboratories, and there were no procedures for conducting these assessments.

#### Quality control and EQA

Despite all laboratories participating in at least one EQA, 63% (15/24) lacked documented procedures to support proficiency testing, and 46% (11/24) laboratories did not conduct root cause analysis or implement CAPA for deviations identified in EQA. Additionally, a comprehensive quality control programme was absent in 42% (10/24) of laboratories. Internal quality control using control strains was either not performed or not documented in 33% (8/24) of laboratories. Furthermore, 38% (9/24) did not conduct quality control checks for each new lot of susceptibility disks, reagents and consumables before use.

#### Quality of water

Water quality was a critical concern, with 71% (17/24) of laboratories produced deionized/distilled water, However, 76% (13/17) did not test the water for purity.

#### Equipment calibration and maintenance

Equipment, calibration and maintenance were other critical areas of concern. Equipment validation, calibration and maintenance were critical areas of concern. In 33% (8/24) of laboratories, equipment was not validated onsite upon installation and before use. Additionally, 46% (11/24) did not adequately evaluate, calibrate, or maintain essential equipment. SOPs for laboratory equipment, including instructions for use and schedules for routine inspection, cleaning, maintenance, testing and calibration, were absent in 54% (13/24), and routine preventive maintenance was not conducted in these facilities.

#### Specimen collection and handling

Specimen collection and handling practices also showed deficiencies. Among the 24 laboratories surveyed, 58% (14/24) collect specimens onsite. Four of these 14 laboratories lacked, however, a procedure for specimen collection.

#### Inventory management

Procedures for the reception, storage, acceptance testing and inventory management of reagents and consumables were absent in 42% (10/24) laboratories. Inventory records were not maintained in 25% (6/24). Furthermore, 25% of the laboratories reported that storage areas were not properly set up or monitored for appropriate conditions, such as temperature and humidity.

#### Electronic systems and IT

**Figure 2.**
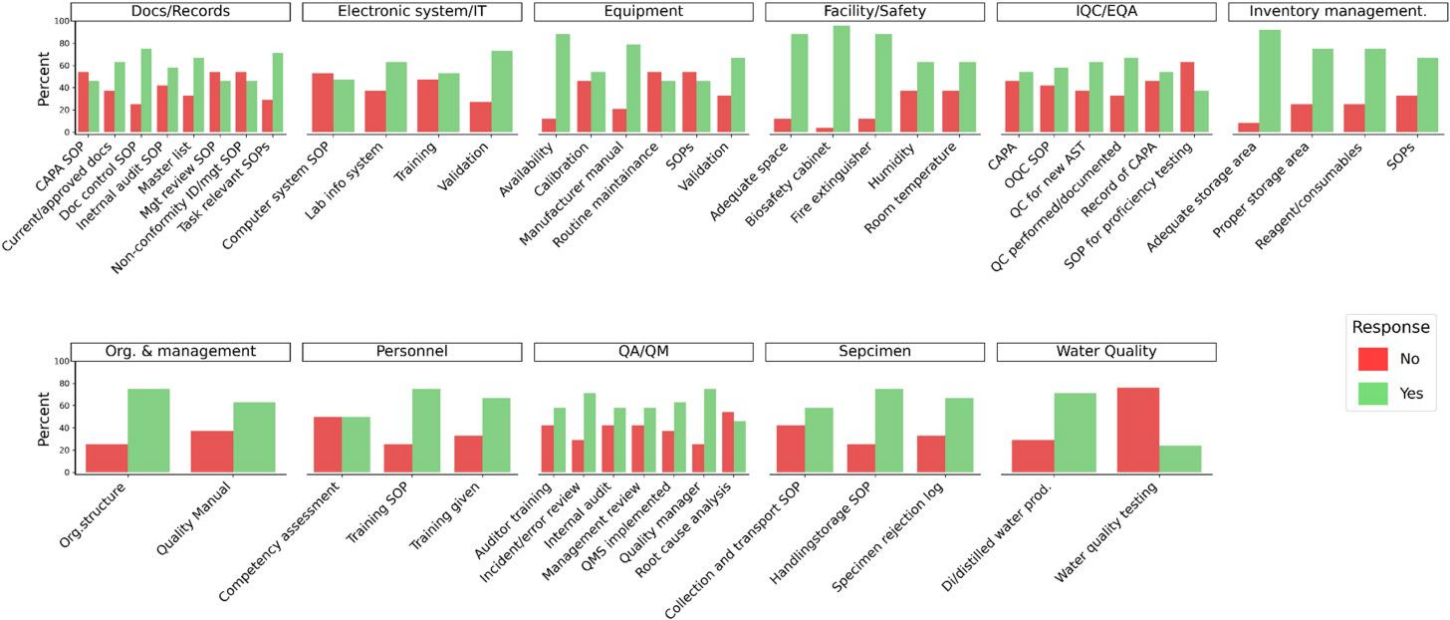
Responses of laboratories participated in the baseline assessment.

Among the 24 laboratories surveyed, 63% (15/24) utilized a laboratory information system (LIS) for managing examination data. However, 27% (4/15) had not validated the LIS software, 53% (8/15) lacked SOPs for LIS, operation and maintenance, as well as corresponding training manuals. Additionally, LIS-specific training for employees was not provided in 47% (7/15) (Figure 2).

### Identified gaps during baseline assessment and improvements observed after follow-up evaluations

Of the four laboratories that completed the baseline survey and were later identified as underperforming in the EQA iterations, three underperformed in two different iterations, and one underperformed in three different iterations. Key gaps included two laboratories had not implemented QMS and had not designated a quality manager to oversee laboratory operations. Additionally, three laboratories did not routinely review error records or incident reports to identify trends and initiate CAPA. Environmental conditions, including room temperature and humidity, were not monitored in two laboratories.

A document control programme was absent in two laboratories to ensure that only the latest versions of documents were in use and in three laboratories, not all documents were controlled, current and approved by authorized personnel. Furthermore, two laboratories did not have SOPs for key processes such as media preparation, pathogen identification and AST testing.

Procedure for staff training was absent, and three laboratories did not provide training on assigned tasks. Furthermore, none of the assessed laboratories conducted competency assessments for their personnel.

Internal quality control was not conducted, and a quality control programme was absent in two laboratories. Root cause analysis and corrective actions for EQA deviations or routine laboratory activities were not implemented in two laboratories. Although three laboratories produced deionized/distilled water, no laboratory tested its purity, and two did not perform quality control checks on a new lot of reagent and consumable.

The most significant gaps identified across all laboratories were related to equipment. None of the laboratories validated equipment upon installation or prior to use. No laboratory had developed or maintained procedures for equipment operation, maintenance and calibration. Additionally, three laboratories did not calibrate equipment used for measurement and testing and routine preventive maintenance of equipment was not conducted in any laboratory.

After the follow-up support provided to the four laboratories, several improvements have been observed. Both laboratories have initiated the implementation of a QMS, and a quality manager has been assigned to oversee laboratory operations and quality management activities. Additionally, environmental conditions, including room temperature and humidity, are now monitored, controlled and recorded in these laboratories.

A document control programme has been established in all surveyed laboratories, and SOPs for key processes have been developed and maintained. Procedures for staff training are available in three laboratories, with staff training on assigned tasks now provided. However, competency assessments have not yet been conducted in these laboratories.

**Figure 3.**
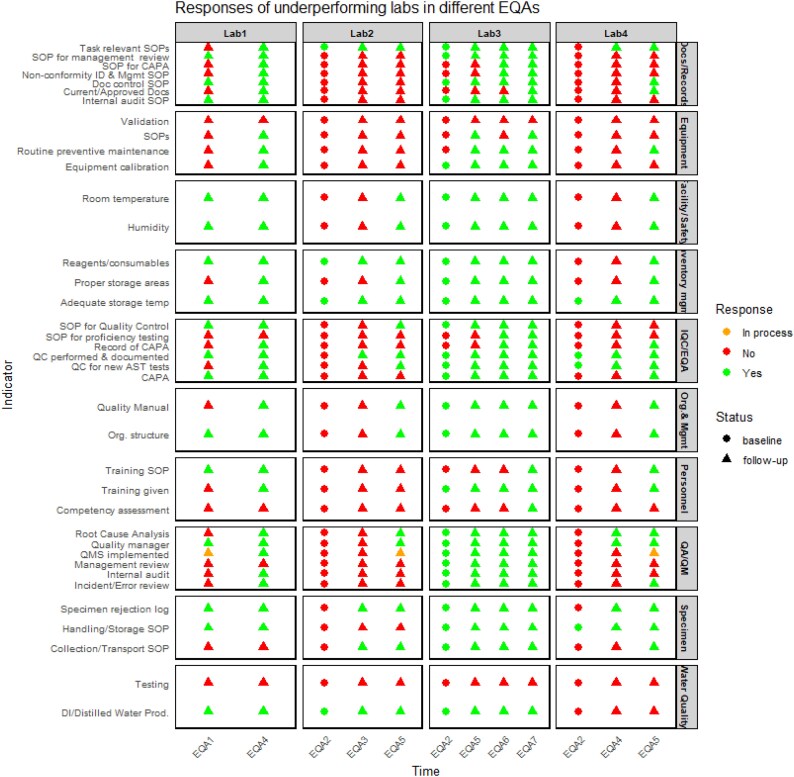
Responses of underperforming laboratories in different EQA round.

Improvements were also observed in quality control. Two laboratories have developed and maintained a quality control programme, and root cause analysis and corrective actions for EQA deviations and routine laboratory activities are now performed and documented in these laboratories. Quality control checks for new lots of susceptibility disks, reagents and consumables are performed in all laboratories. Additionally, procedures for the operation, maintenance and calibration of equipment are in place in two laboratories, with calibration and routine preventive maintenance now being conducted in these labs (Figure 3).

To address EQA deviations in pathogen identification and AST, 1 h online consultations were held. The table below highlights the identified gaps and subsequent improvements (Table 1).

**Table 1.** Summary of gaps identified by EQA and improvement observed after subsequent online consultations

Lab ID	Initial EQA iteration of underperformance	Gaps identified	Feedback provided	Subsequent EQA iteration of underperformance	Reason for underperformance	Improvement observed
Lab 1	EQA 1	Incorrect zone diameter measurement Media pH not checked No routine purity control Incubator temperature not monitored McFarland standard replaced every 4–5 months	Measure media pH Prepare purity control Use dark background for zone measurement Monitor incubator temperature Replace McFarland standard frequently	EQA 4	Low score in *Acinetobacter spp.* panel	Higher AST score from EQA iteration 5 onwards
Lab 2	EQA 3	Gaps in reference strain testing Errors in the identification *Enterococcus faecalis*	Consultation meeting and onsite visit and training conducted after EQA 5 Test reference strains regularly To avoid misinterpretation of the obtained results, two different staff members should both read and interpret results independently	EQA 5	Gaps in the diagnostics and AST testing of enterococci	Gaps resolved after EQA iteration 5
Lab 3	EQA 5	Gaps in diagnostics and AST for *Streptococcus pneumoniae* Gaps in reference strain testing	Consultation meeting Test reference strains regularly To avoid misinterpretation of the results obtained, two different staff members should both read and interpret the results independently Use of appropriate phenotypic tests (i.e. optochin disk, etc.) is important for correct identification	EQA 6 and 7	Gaps in reference strain testing, ID and AST for enterococci	Gaps were resolved after EQA iteration 7
Lab 4	EQA 4	Major gaps in the identification and AST testing across all panel strains	Consultation meeting and onsite visit and training conducted after EQA6 Test reference strains regularly To avoid misinterpretation of the results obtained, two different staff members should both read and interpret the results independently	EQA 5	Identification and AST for enterococci	The laboratory was not underperforming in iterations 6, 7 and 8 The gaps mentioned were not observed then

## Discussion

Quality laboratory testing is critical for detecting and controlling infectious diseases and associated AMR in humans and animals, establishing a cornerstone of effective health interventions. Implementing laboratory QMS, participation in EQA schemes and striving for continuous improvement are essential strategies for enhancing laboratory diagnostics and ensuring reliable and reproducible test results.^14^ Laboratories across South and Southeast Asia face, however, challenges, including limited access to training, and inadequate technical support. To tackle these barriers, the EQASIA project aims to enhance bacteriology diagnostics across the OH sectors in the region by providing comprehensive EQA programmes.

A baseline assessment conducted as part of the EQASIA project revealed several deficiencies in the laboratories’ QMS practices. While implementing a QMS is pivotal in reducing errors and enhancing the efficiency, quality and reliability of laboratory operations,^15^ sustained success requires continuous monitoring through regular internal and external audits to ensure its effectiveness and compliance with international standards.^16^ A baseline assessment revealed that although 13 out of 24 surveyed laboratories had established a QMS, 10 laboratories had not conducted internal audits. This finding highlights a significant gap in the routine, systematic evaluation required for the continuous improvement and sustainability of laboratory operations and the overall effectiveness of the QMS.

Additionally, EQA schemes play a vital role in improving laboratory testing quality and performance over time. It is important to note that participation alone is insufficient to ensure sustained improvement. Consistent performance enhancement requires systematically identifying and addressing the root causes of errors that contribute to suboptimal outcomes.^17,18^ The baseline assessment further indicated that 46% of the laboratories did not conduct root cause analysis or implement CAPA for deviations identified through EQA or routine laboratory activities. This lack of thorough analysis and an effective CAPA process contributes to the recurrence of errors, hindering long-term improvements in laboratory performance.^16^

A major component of the QMS is internal quality control, which is routinely conducted to monitor the precision and accuracy of test procedures, the performance of reagents, and equipment.^19^ Our findings revealed gaps in the implementation of internal quality control, including inconsistent use of control strains, which are crucial for verifying test accuracy. Furthermore, laboratory water used in microbiological analyses should be routinely tested to ensure acceptable bacterial plate count levels (cfu/ml), as the quality of culture media is heavily influenced by the quality of water.^20^ Among the 17 laboratories producing distilled or deionized water, only three tested their water for purity, potentially compromising the accuracy of test results.

In addition to internal quality control, the reliability of laboratory results linked to the skills and competence of laboratory staff. Insufficient training and competency assessments can compromise staff performance and the quality of laboratory output. To ensure accuracy and consistency, laboratories must develop and maintain SOPs, implement structured competency assessment programmes and perform regular equipment calibration and maintenance.^21^ Our study identified substantial gaps in these areas. Training on assigned tasks was inconsistent, and competency assessments were not routinely performed. Furthermore, SOPs for key processes such as media preparation, pathogen identification and AST testing were missing possibly due to insufficient training in document preparation. Equipment management also showed deficiencies, as essential lab equipment, including micropipettes, thermometers, centrifuges, balances, water baths and incubators, was not regularly tested, calibrated, or maintained as per standards. This lack of routine equipment management increases the risk of inaccurate results, compromising the reliability of the laboratory.

Participation in EQA programmes, combined with targeted follow-up supports, contributes to the laboratory improvement and sustained performance.^2^ In a review of underperforming laboratories, several key deficiencies were identified, including pathogen misidentification, misinterpretation of AST results and irregular testing of reference strains. Contributing factors included deviations from the CLSI guidelines such as using non-recommended antibiotic concentrations (e.g. 10 µg cefoxitin for AST) and the absence of robust quality control measures, such as independent verification of results by two members of staff. Additionally, the failure to routinely test reference strains compromised the ability to ensure test systems were functioning correctly and producing results within specified limits. Errors in pathogen identification were further worsened by the lack of appropriate phenotypic tests, such as the use of optochin disks for identifying *Streptococcus pneumoniae*.

After EQA participation, targeted online consultations, guidance and onsite visits, substantial improvements were observed in both the QMS and technical operation of underperforming laboratories. Notable progress included the initiation of QMS implementation in underperforming laboratories, the establishment of document control systems and the development of SOPs for key processes such as media preparation, pathogen identification and AST. These initiatives reflect efforts by laboratories to provide quality laboratory service and align with best practices.

Further progress was observed in the implementation of staff training programmes, quality control measures and the implementation of root cause analysis and CAPA process. This demonstrates a shift towards a proactive approach to quality assurance. Additionally, improvement in equipment management such as calibrating equipment, establishing preventive maintenance programmes and developing procedures for equipment operation, maintenance and calibration highlight progress in improving the accuracy and reliability of laboratory operations.

Laboratory assessments and follow-up support for underperforming laboratories were primarily conducted through survey questionnaires and online meetings. However, the inability to conduct onsite visits and training sessions for all underperforming laboratories, due to resource constraints COVID-19-related restrictions, security issues at the country level posed a significant challenge. Despite these challenges, the observed improvements highlight the value of targeted follow-up support in strengthening laboratory capacity and ensuring continuous performance enhancement.

In conclusion, our findings demonstrated the critical role of EQA participation, coupled with targeted follow-up support, guidance and onsite training, in enhancing the quality of laboratory operations. Strengthening and ensuring the sustainability of EQA schemes and targeted follow-up support are essential for the continuous improvement of laboratory capacity in bacterial identification and AST testing. These improvements ultimately contribute to the provision of reliable data that can guide policy actions to combat AMR.

## Supplementary Material

dkaf266_Supplementary_Data
