# Supplementary material for: Strengthening antimicrobial resistance diagnostics in National Reference Laboratories across One Health in South and Southeast Asia: impact of external quality assessment and targeted follow-up
Source: J Antimicrob Chemother. 2025 Jul 29;80(9):2541–9. doi: 10.1093/jac/dkaf266 (PMC12404728; doi:10.1093/jac/dkaf266)
Supplement: dkaf266_Supplementary_Data [file dkaf266_supplementary_data.doc]

| LABORATORY FOLLOW UP visit ASSESSMENT TOOL | |
| --- | --- |
| **Laboratory Name/Address** |  |
| **Laboratory Contact**   - **Contact Name** - **Phone Number** - **Email** |  |
| **Visit Date(s)** |  |
| **Visit Location (Country or Region)** |  |
| *.*   - **Visit Standards:** 21 CFR 11, 21 CFR 312/812, 42 CFR 493, College of American Pathologist (CAP) Checklists and CLSI (NCCLS) Guidelines (As applicable), ISO17025 and ISO15189 - **Regardless of laboratory designation (i.***e., clinical/medical, animal and food safety laboratory etc.) all phases of laboratory analysis will be evaluated as follows (Note: A failure of any one of these critical phases of clinical laboratory testing can significantly affect the integrity of a clinical result):*   - Preanalytical Phase (Pre-examination Phase) - Preanalytical variables monitored*, i.e., pre-examination, variables include all steps in the process prior to the analytic phase of testing, starting with the order received in the lab (e.g., by physician, GCP personnel, etc.). Examples include (Note: This list is neither all-inclusive nor exclusive. The variables chosen should be appropriate to the laboratory's scope of care):*     - *Accuracy of transmission of lab orders,*     - *specimen transport and preparation,*     - *specimen chain of custody,*     - *requisition accuracy,*     - *quality of phlebotomy services,*     - *specimen acceptability rates (acceptance/rejection),*     - *analytic method and electronic system validation,*     - *equipment calibration and maintenance*   - Analytic Phase (Examination Phase) *– Analytic Phase represents all variables that can impact sample integrity and analysis during the testing phase. Examples include (Note: This list is neither all-inclusive nor exclusive. The variables chosen should be appropriate to the laboratory's scope of care):*      - *Preparation of slides, solutions, calibrators, controls, reagents*     - *proficiency testing materials,*     - *Interfering substances (e.g., silicates in lab water, detergent residues related to glassware washing).*     - *quality of water and other materials used in testing*     - *Ensuring expected results are defined prior to analysis and may include defined reportable ranges for test results (i.e., normal values)*     - *References to manufacturer's test system instructions, package inserts and operator manuals*     - *Identification of panic or alert values (as applicable)*   - Post-Analytical Phase (Post-Examination Phase) *- Post analytic variables monitored, i.e., post-examination, variables include all steps in the overall laboratory process between completion of the analytic phase of testing and results receipt by the requesting physician. Examples include (Note: This list is neither all-inclusive nor exclusive. The variables chosen should be appropriate to the laboratory's scope of care):*     - *review, approval and release of result reports and corrected reports*     - *accuracy of data transmission across electronic interfaces,*     - *monitoring of turnaround time from test completion to chart posting (paper and/or electronic),*     - *interpretability of reports*     - *post-analysis sample storage and result retention* | |

| **EVALUATION FOR:** | |
| --- | --- |
| **Inclusive List of Documents Reviewed** | |
| **Version Date** | **Document Title** |
|  |  |
|  |  |
|  |  |
|  |  |
|  |  |
|  |  |
|  |  |
|  |  |
|  |  |
|  |  |
|  |  |
|  |  |
|  |  |
|  |  |
|  |  |
|  |  |
|  |  |
|  |  |
|  |  |
|  |  |
|  |  |
|  |  |
|  |  |
|  |  |

| **ASSESSMENT QUESTIONS** | **Yes** | **No** | **N/A** | **Supporting Explanation** |
| --- | --- | --- | --- | --- |
| **A. Facility and Security** |  |  |  |  |
| 1. Is access to the laboratory restricted? |  |  |  |  |
| 1. How is entry monitored and restricted? |  |  |  | Do you have access control for entry in your laboratory? |
| 1. Is security staff available 24 hours a day? |  |  |  |  |
| 1. What are the backup services if a power failure occurs? |  |  |  |  |
| 1. Does the laboratory have separate room for specimen collection, media preparation, sample preparation of microbiological analysis and reference testing? |  |  |  |  |
| 1. Where a Biosafety cabinet is required to perform work, is it certified and appropriate? |  |  |  |  |
| 1. Are environmental conditions checked and recorded daily (room temperature, freezer, refrigerator, incubators, water bath)? |  |  |  |  |
| 1. Is an appropriate fire extinguisher available, properly placed, in working condition, and routinely inspected? |  |  |  |  |

| **ASSESSMENT QUESTIONS** | **Yes** | **No** | **N/A** | **Supporting Explanation** |
| --- | --- | --- | --- | --- |
| **B. Organization and Management** |  |  |  |  |
| 1. What type of testing does the laboratory do? (Scope of operations when your laboratory was established?) |  |  |  |  |
| 1. Is there a current laboratory quality manual that is implemented within your organization? |  |  |  |  |
| 3. Do your organizational charts show:   - the organization and management structure of the laboratory, - The laboratories place in any parent organization, - The relations between the laboratory, management, technical operations, support services and the quality management system. |  |  |  |  |
| 1. Who in the laboratory has overall responsibility for the technical operations and the provision of the resources needed to ensure the required quality of laboratory operations? |  |  |  |  |
| Other: |  |  |  |  |

| **ASSESSMENT QUESTIONS** | **Yes** | **No** | **N/A** | **Supporting Explanation** |
| --- | --- | --- | --- | --- |
| **C. Personnel, Orientation, Training, and Assessment** |  |  |  |  |
| 1. How many personnel does your lab employ? |  |  |  |  |
| 1. Is there a training SOP defining processes for new employee training and orientation, internal and external training? |  |  |  |  |
| 1. Does each employee receive training on new and updated procedures and policies, quality management systems, assigned work processes, procedures, and tasks? |  |  |  |  |
| 1. Is this documented in writing to include dates of training, type of training, and the signature of the employee and trainer? |  |  |  |  |
| 1. Please describe how your laboratory assesses and documents employee competency on the tasks they routinely perform (i.e., supervisor, consultant, and testing personnel) |  |  |  |  |
| 1. Are training records readily retrievable in a manner that enables one to determine what training an employee has received, which employees have been trained on a particular procedure, or have attended a particular training program? |  |  |  |  |
| 1. Are records of personnel files which include job description, job orientation, previous work experience (CV), competency assessment, review of staff performance, education, personnel qualification, etc. maintained? |  |  |  |  |

**Employee Training and Qualification – Select at least five (5) employee files from different functional areas for review**

| Name | Title | Hire Date | Training Records |
| --- | --- | --- | --- |
|  |  |  |  |
|  |  |  |  |
|  |  |  |  |
|  |  |  |  |
|  |  |  |  |
|  |  |  |  |
|  |  |  |  |

| **ASSESSMENT QUESTIONS** |  | | | **Supporting Explanation** |
| --- | --- | --- | --- | --- |
| **D. Quality Assurance/Quality Management** | **Yes** | **No** | **N/A** |  |
| *The laboratory must have a documented quality management system to systematically evaluate the quality and appropriateness of laboratory services. The program must be designed to identify and resolve important problems in patient care and identify opportunities to improve patient care.* | | | | |
| 1. QM - Does the laboratory have a documented quality management (QM) system? |  |  |  |  |
| 1. QM - Does the quality management (QM) program follow a documented operational plan (This plan may be based upon some reference resource such as CLSI GP-22 ISO15189 or ISO17025 series, for improving organizational performance)? |  |  |  |  |
| 1. QM- Does the laboratory summarize and review its records of errors and incident reports at defined intervals to identify trends and initiate corrective and preventive actions (CAPA) as appropriate (Complaints follow up and trending at defined intervals)? |  |  |  |  |
| 1. QM – Are Preanalytical (e.g., order processing, specimen collection, transport), Analytical (e.g., sample receipt, processing, QC), and Post Analytical (e.g., turn-around-times and result reports) variables monitored? |  |  |  |  |
| 1. QM- Is the QM program reviewed at least annually for effectiveness (There must be documentation that the laboratory director or designee(s) reviews the program regularly. |  |  |  |  |
| 1. Is there a dedicated quality assurance department that is responsible for monitoring the laboratory to assure management that the facilities, equipment, personnel, methods, practices, records, and controls are in conformance with the regulations? |  |  |  |  |
| 7. Does a formal auditing function exist in the QA department and is defined by SOPs to ensure all activities of the quality management system are audited? |  |  |  |  |
| 8. Is internal auditing performed to confirm that all activities in the SOPs of the laboratory are being followed as written? |  |  |  |  |
| 9. Does the quality SOP specify who conducts audits? |  |  |  |  |
| 10. Are the personnel conducting the internal audits trained with proven competency in auditing managerial and/or technical requirements? |  |  |  |  |
| 11. Does the quality SOP specify the scope and frequency of audits and how such audits are to be documented? |  |  |  |  |
| 12. Are internal audit findings presented to the laboratory management and relevant staff for review? |  |  |  |  |
| 13. Is documented root cause analysis performed for non-conforming work before corrective actions are implemented? |  |  |  |  |
| 14. Does a formal Corrective and Preventive Action (CAPA) procedure exist to ensure observations identified during routine auditing are associated with Root cause analysis, CAPA, effectiveness verification, and CAPA closure, management, and tracking? |  |  |  |  |

| **ASSESSMENT QUESTIONS** | **Yes** | **No** | **N/A** | **Supporting Explanation** |
| --- | --- | --- | --- | --- |
| **E. Doc Control/Standard Operating Procedures (SOP)** |  | | |  |
| 1. Is there a documented process for creating, reviewing, updating SOPs? |  |  |  |  |
| 1. Are policies and/or SOPs for laboratory functions, available, current, and approved by authorized personnel? |  |  |  |  |
| 1. Are all quality management procedures, forms, and records maintained under document control? |  |  |  |  |
| 1. Is there a documented process for ensuring that all staff are trained on pertinent SOPs and receive refresher training when SOPs are updated? |  |  |  |  |
| 1. How are SOPs handled when they are no longer needed? |  |  |  |  |
| 1. Are all SOPs established by the authority of the management? Is a historical life of SOPs, and all revisions, including the dates of such revisions maintained? |  |  |  |  |
| 1. Does each laboratory area have immediately available laboratory manuals and SOPs relative to the laboratory procedures being performed (i.e., SOP for media preparation, pathogen identification and AST)? |  |  |  |  |
| 1. Is there an archiving system that allows for easy and timely retrieval of archived records and results? |  |  |  |  |

| **ASSESSMENT QUESTIONS** | **Yes** | **No** | **N/A** | **Supporting Explanation** |
| --- | --- | --- | --- | --- |
| **F. Method Validation** | **IF THIS SECTION DOES NOT APPLY TO YOUR LAB PLEASE ENTER “N/A” HERE** | | |  |
| *CLINICAL/MEDICAL LAB REQUIREMENTS*  *NOTE: The reportable range includes all results that may be reliably reported, and embraces two types of ranges:*   1. *The ANALYTICAL MEASUREMENT RANGE (AMR) is the range of analyte values that a method can directly measure on the specimen without any dilution, concentration, or other pretreatment not part of the usual assay process* 2. *The CLINICALLY REPORTABLE RANGE (CRR) is the range of analyte values that a method can measure, allowing for specimen dilution, concentration, or other pretreatment used to extend the direct analytical measurement range*   *The limits of the reportable range are based on meeting accuracy and precision requirements such as the minimal limit of quantification or sensitivity, when applicable. In some cases, clinically relevant limits may be narrower than the potential analytical range, and the clinically relevant limit would be used as the limit of the reportable range.* | | | | |
| 1. What is the process for developing a method validation? |  |  |  |  |
| 1. How are issues or amendments that arise with the validation documented and communicated to appropriate departments? |  |  |  |  |
| 1. What is the process for verifying accuracy of the method validation procedure/ |  |  |  |  |
| 1. Does auditing of the validation process take place? |  |  |  |  |
| 1. Who approves and signs the Validation Report? |  |  |  |  |

| **ASSESSMENT QUESTIONS** | **Yes** | **No** | **N/A** | **Supporting Explanation** |
| --- | --- | --- | --- | --- |
| **G. Sample Shipping, Receipt/Processing (Preanalytical)** |  |  |  |  |
| *This section addresses specimens received from remote locations outside of the facility in which the laboratory is located, as well as specimens referred by the laboratory to other locations. While transportation of clinical specimens may not be the responsibility of personnel under the control of the laboratory director, issues of tracking and specimen quality must be addressed to ensure quality laboratory results.* | | | | |
| 1. Is there a dedicated samples or isolates receiving area in your laboratory (i.e., shipping/receiving, and accessioning area)? |  |  |  |  |
| 1. Are samples appropriately stored prior to analysis (i.e., at correct temperatures for sample type, in non-frost-free freezers, in controlled areas of the laboratory)? |  |  |  |  |
| 1. Is there a documented tracking system to ensure that all samples or isolates that were shipped from origin (e.g., hospital, collection site, etc.) were received and registered? |  |  |  |  |
| 4. Are personnel responsible for specimen handling and processing to routinely trained to follow these procedures? |  |  |  |  |
| 5. Is the lab open to receive samples and for testing, beyond regular working hours? Record days/wk, hrs/day |  |  |  |  |
| 6. Are incoming samples adequately identified? |  |  |  |  |
| 7. Upon receipt what document are checked against to samples to ensure that all samples are present? |  |  |  |  |
| 9. Are all samples or isolates accompanied by a test requisition upon delivery to the laboratory? |  |  |  |  |
| 10 How primary samples or isolates stored after initial analysis? |  |  |  |  |
| 11. How does the sample receipt group handle samples that are broken in transit, quantity not sufficient (QNS) or shipped in at the wrong temperature (i.e., acceptance / rejection criteria)? |  |  |  |  |
| 12. How are samples handled that are mislabeled or unlabeled? |  |  |  |  |
| 13. Will sample testing be held up if pertinent information is missing on the test requisition (i.e., demography, test requests, etc.)? |  |  |  |  |
| 14. How is missing information obtained? |  |  |  |  |
| 15. Do you have a specimen rejection logbook? |  |  |  |  |

| **ASSESSMENT QUESTIONS** | **Yes** | **No** | **N/A** | **Supporting Explanation** |
| --- | --- | --- | --- | --- |
| **H. Specimen Collection, Handling and Reporting** |  | | |  |
| *Specimen collection, data handling, and results reporting are critical. Specific instructions for the proper collection and handling of specimens must be made available to laboratory personnel and to anyone collecting patient test materials that are sent to the laboratory.* | | | | |
| 1. Is there a procedure manual or other source for the complete collection and handling instructions of all laboratory specimens? |  |  |  |  |
| 1. Does the director or designee review and approve all changes to the specimen collection/handling procedure manual before implementation? |  |  |  |  |
| 1. Does the specimen collection manual include instructions for all the following elements, as applicable)? Preparation of the patient 2. Type of collection container and amount of specimen to be collected 3. Types and amounts of preservatives or anticoagulants 4. Need for special handling between the time of collection and time received by the laboratory (*e.g.*, refrigeration, immediate delivery) 5. Proper specimen labeling 6. Need for appropriate clinical data, when indicated |  |  |  |  |
| 4. Is there documentation that all personnel performing specimen collection have been trained in the proper selection and use of equipment/supplies, and collection techniques? |  |  |  |  |
| 5. Are specimens uniquely identified to minimize sample mix-ups, mislabeling, etc. (All specimens must be labeled at the time of collection to provide unique identification)? |  |  |  |  |
| 1. Are copies or files of reported results retained by the laboratory in a manner that permits prompt retrieval of the information? |  |  |  |  |
| 1. Are laboratory records and materials retained for an appropriate time? |  |  |  |  |
| 1. Is there a documented protocol in place to ensure that data are accessible only to those personnel who are authorized to review results (e.g., per country specific regulatory requirement) |  |  |  |  |

| **ASSESSMENT QUESTIONS** | **Yes** | **No** | **N/A** | **Supporting Explanation** |
| --- | --- | --- | --- | --- |
| **I. Proficiency Testing** |  | | |  |
| 1. Are the laboratory’s procedures for proficiency testing written and sufficient for the extent and complexity of testing done in the laboratory? To included PT that was intended to be graded but was not? |  |  |  |  |
| 1. Is there appropriate documentation of problems and their solutions identified by the proficiency testing system? (e.g., Corrective and preventive action) |  |  |  |  |
| 1. Is there a policy that prohibits interlaboratory communication about proficiency testing samples until after the deadline for submission of data to the proficiency testing provider? |  |  |  |  |
| 1. Is there a policy that prohibits referral of proficiency testing specimens to another laboratory and interlaboratory communication? Test not performed if reference lab used |  |  |  |  |
| 1. How often does your laboratory participate in EQA for AST? |  |  |  |  |
| 1. If deviating results are obtained in EQA what follow-up done/ measure is taken to address the nonconformance? |  |  |  |  |
| 1. Is there written evidence that all problems identified by proficiency testing and alternative performance assessment have been recognized and corrected? |  |  |  |  |

| **ASSESSMENT QUESTIONS** | **Yes** | **No** | **N/A** | **Supporting Explanation** |
| --- | --- | --- | --- | --- |
| **J. Glassware/Quality of Water/Reagents** |  | | |  |
| *NOTE:* *The laboratory should define the type of water necessary for each of its procedures and should have an adequate supply of same. Reagent grades, as defined by the CLSI Guideline C3‑A3 include the following specifications at time of production:*   |  | *Type I* | *Type II* | *Type III* | | --- | --- | --- | --- | | *Maximum microbial content (CFU/mL)* | *10* | *1000* | *n/a* | | *Minimum resistivity (megohm-cm)* | *10 (in-line)* | *1.0* | *0.1* | | *Maximum silicate content (mg/L SiO2)* | *0.05* | *0.1* | *1.0* | | *Particulate matter* | *0.22 um filter* | *n/a* | *n/a* |   *Bacteria may inactivate reagents, contribute to total organic contamination, or alter optical properties of test solutions. Resistivity provides a nonspecific measure of the ion content. Silicates or colloidal silica may interfere with certain assays.* | | | | |
| 1. Is glassware routinely inspected for cracks, chips, etc.? |  |  |  |  |
| 1. What is the process for ensuring that glassware is clean and free of contaminants and detergent residues? |  |  |  |  |
| 1. Is reagent grade water prepared? |  |  |  |  |
| 1. Is the reagent grade water tested for purity, etc.? |  |  |  |  |
| 1. Is there a documented statement of policies and procedures that defines the standards for, and frequency of testing water quality? |  |  |  |  |

| **ASSESSMENT QUESTIONS** | **Yes** | **No** | **N/A** | **Supporting Explanation** |  |
| --- | --- | --- | --- | --- | --- |
| **K. Equipment Calibration and Maintenance** |  | | |  |  |
| 1. Does the laboratory have appropriate equipment available for performing pathogen identification and AST? |  |  |  |  |  |
| 1. Is back up equipment available if a piece of equipment fails? |  |  |  |  |  |
| 1. Are all equipment and methods validated/verified on-site upon installation and before use and is documented evidence available? |  |  |  |  |  |
| 1. Do the SOPs for equipment describe remedial action to be taken in the event of failure or malfunction? |  |  |  |  |  |
| 1. Are the manufacturer’s operator manuals readily available to testing staff and, available in the language understood by staff? |  |  |  |  |  |
| 1. Is equipment used for measurement, testing or assessment of data adequately tested, calibrated, and/or maintained? |  |  |  |  |  |
| 1. Do the SOPs for equipment contain sufficient detail regarding methods, materials, and schedules to be used in routine inspection, cleaning, maintenance, testing, calibration of equipment, and designate the person responsible for the performance of each operation? |  |  |  |  |  |
| 1. Does the laboratory have a calibration procedure for calibration dependent equipment (pipettes, centrifuges, balances, and thermometers)? |  |  |  |  |  |
| 1. Is current equipment inventory data available for all equipment in the laboratory including the following information?  - Name of equipment - Manufacturers or authorized supplier contact details - Condition received (new, used, reconditioned) - Serial number - Date of receiving - Date of entry into service after validation/ verification |  |  |  |  |  |
| 1. Are equipment operated by trained, competent and authorized personnel? |  |  |  |  |  |
| 1. Is non-functioning equipment appropriately labelled and removed from the laboratory or path of workflow following the equipment management policies and procedures? |  |  |  |  |  |

**Complete Table for all calibration dependent equipment sampled**

| **EVALUATION FOR:** | |
| --- | --- |
| **Equipment Calibration Review** | |
| **Device** |  |
|  |  |
|  |  |
|  |  |
|  |  |

| **ASSESSMENT QUESTIONS** | **Yes** | **No** | **N/A** | **Supporting Explanation** |
| --- | --- | --- | --- | --- |
| **L. Quality Control (QC)** |  | | |  |
| *The overall quality control program for the entire laboratory must be clearly documented. It must include general policies and delegation of responsibilities. The quality control records should be well‑organized with a system to permit regular review by appropriate supervisory personnel (laboratory director, supervisor, or laboratory quality control coordinator).* | | | | |
| 1. Is there a written QC program that defines procedures for determining analytic performance, establishments of limits, frequency of controls, and corrective action for QC data? |  |  |  |  |
| 2. Which guidelines are used for the interpretation of Antimicrobial Susceptibility Testing? |  |  |  |  |
| 3. Are Antimicrobial susceptibility Testing guidelines regularly updated? |  |  |  |  |
| 4. Is internal quality control performed, documented, and verified for pathogen identification and AST tests/procedures before releasing patient results? |  |  |  |  |
| 5. Does the laboratory use specific bacterial control strains to assure the quality of media for bacterial cultures? |  |  |  |  |
| 6. How frequently does the laboratory test Quality Control strains? |  |  |  |  |
| 7. Do you store the EQA strains for later reference? |  |  |  |  |
| 8. How is quality control reference strains handled and stored? |  |  |  |  |
| 9. What is the Quality Control procedure to ensure purity of the control plate? |  |  |  |  |
| 10. Are Quality Control records are available? |  |  |  |  |
| 11. At what interval does the Quality Control records reviewed? |  |  |  |  |
| 12. What remedial actions are taken if unacceptable errors are discovered during review? |  |  |  |  |
| 13. Does your laboratory perform quality control for antimicrobial discs using control strains before placing newly received lot numbers/shipments into use? |  |  |  |  |

| **ASSESSMENT QUESTIONS** | **Yes** | **No** | **N/A** | **Supporting Explanation** |
| --- | --- | --- | --- | --- |
| **M. Inventory Management** |  | | |  |
| 1.Does the laboratory have a procedure for the reception, storage, acceptance testing and inventory management of reagents and consumables. |  |  |  |  |
| 2. Does the lab maintain records for each reagent and consumable that contributes to the performance of examinations? (e.g., identity of the reagent, batch code or lot number, date of receiving, expiry date, date of entering service etc..) |  |  |  |  |
| 3. Are inventory records complete and accurate, with minimum and maximum stock levels denoted and monitored? |  |  |  |  |
| 4. Is adequate cold storage available? |  |  |  |  |
| 5. Are storage areas set up and monitored appropriately (temperature, humidity)? |  |  |  |  |
| 6. Are storage areas access-controlled? |  |  |  |  |
| 7. Are all reagents/test kits in use (and in stock) currently within the manufacturer-assigned expiration or within stability? |  |  |  |  |
| 8. Is each new reagent preparation, new lot number, new shipment of reagents or consumables verified before use and documented? |  |  |  |  |
| 9. Is First Expiration-First-Out (FEFO) practiced? |  |  |  | Do you store products that will expire first in front of products with a later expiry? |

| **ASSESSMENT QUESTIONS** | **Yes** | **No** | **N/A** | **Supporting Explanation** |
| --- | --- | --- | --- | --- |
| **N. Sample Storage and Inventory** |  | | |  |
| 1. Is there a sample storage tracking and inventory system? |  |  |  |  |
| 1. How are samples logged/tracked that are placed in storage? |  |  |  |  |
| 1. Are freezer and refrigerator temperatures monitored continuously**?** |  |  |  |  |
| 1. How is it determined what samples are available for assay? |  |  |  |  |
| 1. What is the process for assuring that the freezers, refrigerators, etc. are properly maintained? |  |  |  |  |

| **ASSESSMENT QUESTIONS** | **Yes** | **No** | **N/A** | **Supporting Explanation** |
| --- | --- | --- | --- | --- |
| **O. Electronic Systems and IT** | IF YOU DO NOT HAVE AN LIS or LIMS SYSTEM THEN THIS SECTION IS NOT APPLICABLE | | |  |
| 1. Which method (e.g., manual/digital/preprinted result sheet) does your laboratory used to register AST result |  |  |  |  |
| 1. Is a computer system used to manage and report results? |  |  |  |  |
| 1. Is the computer system validated? |  |  |  |  |
| 1. Is an individual identified as responsible for the computer system? |  |  |  |  |
| 1. Is there security in place to limit access to all computer applications and prevent intrusions? |  |  |  |  |
| 1. Do SOPs exits for all uses, operation, and maintenance of the computer system? |  |  |  |  |
| 1. Is there a training manual for all computer applications? |  |  |  |  |
| 1. Is there documented training for all staff on the use of all computer systems? |  |  |  |  |

**GENERAL COMMENTS:**

**LIMS SYSTEM**

**PROFICIENCY TESITING**

**PROJECT MANAGEMENT**


**LIST OF OBSERVATIONS:**

Signed Auditor Name and Date
